# Household costs and health-related quality of life of childhood multidrug-resistant TB in Western Cape, South Africa

**DOI:** 10.5588/ijtldopen.25.0616

**Published:** 2026-07-13

**Authors:** T. Wilkinson, A.C. Hesseling, G. Hoddinott, M.G. Anthony, H.S. Schaaf, E. Sinanovic, J.A. Seddon

**Affiliations:** 1 Health Economics Unit, School of Public Health, University of Cape Town, Cape Town, South Africa;; 2 Desmond Tutu TB Centre, Department of Paediatrics and Child Health, Stellenbosch University, Cape Town, South Africa;; 3 School of Public Health, Faculty of Medicine and Health, University of Sydney, Camperdown, NSW, Australia;; 4 Department of Infectious Disease, Imperial College London, London, UK.

**Keywords:** tuberculosis, socio-economic status, paediatric, health-related quality of life, MDR-TB

## Abstract

**BACKGROUND:**

Multidrug-resistant TB (MDR-TB) in children remains a major public health challenge. Although treatment is free of direct charge in many countries, it can impose substantial indirect and non-medical costs on affected households. Evidence on the economic burden of childhood MDR-TB on families remains limited.

**METHODS:**

A cross-sectional household survey was conducted in the Western Cape, South Africa, among 45 households with a child <15 years who initiated MDR-TB treatment between 2018 and 2021. Socio-economic status, costs of accessing care, and health-related quality of life (HRQoL) were assessed and linked to health service utilisation data to estimate household-level costs.

**RESULTS:**

The median total cost per household was ZAR 7,443 (US$504) per episode of care (interquartile range: ZAR 4,119–13,207), with indirect costs accounting for the largest share of household costs. Twenty-three (51.1%) of households incurred catastrophic health expenditure, defined as >20% of annual household income. Costs increased with hospital-based care, longer treatment duration, and more frequent caregiver visits. The HRQoL of children was generally high, though not uniformly distributed.

**CONCLUSION:**

Childhood MDR-TB places substantial financial burden on already vulnerable households. Economic evaluations and care models should incorporate household costs and consider strategies to reduce the indirect burden of treatment on families.

Multidrug-resistant TB (MDR-TB) is defined as disease caused by *Mycobacterium tuberculosis* that is resistant to isoniazid and rifampicin. Globally, an estimated 400,000 individuals develop MDR-TB each year, of whom an estimated 30,000 are children (<15 years).^1^ Treatment of MDR-TB is more complicated than drug-susceptible TB (DS-TB) and involves the use of more toxic, and frequently more expensive second-line drugs that typically must be taken over a longer duration. In addition to health-system costs, it is critical to understand the costs that are incurred by families for accessing care when a child family member is treated for MDR-TB.^1,2^

Although promising research continues to advance the development of shorter MDR-TB treatment regimens and formulations to improve patient experience and outcomes, the available literature on the costs and socio-economic status (SES) of households with children receiving TB care is sparse. Atkins et al.^3^ conducted a scoping review of studies assessing the socio-economic impact of TB in children. While the review identified four qualitative studies focused on the socio-economic impacts of MDR-TB in children, it did not find any quantitative studies. Shah et al.^4^ conducted an extensive SES survey of households where there was a child with DS-TB but excluded households with children receiving treatment for MDR-TB given the heterogeneity of treatment pathways and patient experience of DS-TB and MDR-TB.^4^ Given that TB disproportionately affects impoverished communities, even minor expenses associated with accessing care can pose substantial barriers for vulnerable households. The absence of data on the financial burden faced by households affected by childhood MDR-TB limits full economic assessment of interventions and treatment modalities, a substantial gap to inform policy. Household surveys can provide granular and localised insights into patient and household experiences of illness across various domains, such as household characteristics, incurred costs, and health-related quality of life (HRQoL).^5^

In 2019, the COVID-19 pandemic spread globally and caused substantial disruptions in health care access and delivery across various disease areas. Disruptions to TB services impacted on diagnosis, treatment, and prevention, with marked increases in disease burden that have persisted for several years post the pandemic in South Africa and in other settings.^6,7^ In addition to better understanding the cost for families of children being treated for MDR-TB, it is also important to understand the impact on these costs of health-system shocks like the COVID-19 pandemic.

## METHODS

South Africa has a high burden of TB, with a total TB incidence of 427 per 100,000 population and an estimated 13,000 people with incident MDR-TB in 2023.^1^ South Africa also faces high rates of child poverty. In 2022, 7.9 million children were living below the food poverty line, and 32% resided in households where no adult was employed.^8^ These households commonly rely on a mix of informal employment and other economic activity, support networks, and government grants for income.^9^ In South Africa, MDR-TB diagnosis and treatment, particularly in children requiring specialist care, are predominantly delivered within the public health sector. The study was conducted in Cape Town, Western Cape, South Africa, at a major public-sector MDR-TB treatment site serving a large urban catchment population. The Western Cape is one of nine provinces in South Africa, with a population of approximately 7.4 million people. The province is linguistically diverse, with Afrikaans (approximately 41.2%), isiXhosa (approximately 31.4%), and English (approximately 22%) being the most commonly spoken home languages.^10^ The province includes both urban and peri-urban communities and exhibits marked socio-economic inequality, with substantial variation in household income, housing infrastructure, and employment status.

### Study design

A descriptive, cross-sectional household survey was conducted to collect data from households regarding the treatment and impact of childhood MDR-TB. Three survey modules were developed: i) household SES and employment (including income and employment related to the COVID-19 pandemic), ii) out-of-pocket expenses for accessing care, and iii) HRQoL. Survey instruments were developed for both paper-based and REDCap digital tools on tablets and were created in isiXhosa, Afrikaans, and English, and participants were matched with researchers fluent in their preferred language.

### Household survey development

The survey was originally adapted from the World Health Organization (WHO) Patient Cost Surveys Handbook (2017)^11^ and a Tuberculosis Control Assistance Program instrument that collected SES information and household expenses of adults with TB (2008).^12^ Substantial modifications were made, incorporating additional components to align with the study objectives. To collect HRQoL data, permission was gained from the EuroQol group for use and translation of the EQ-5D-Y-3L1^13^ instrument into isiXhosa and Afrikaans, and for use of the Toddler and Infant (TANDI) instrument^14^ (subsequently renamed the ‘EQ-TIPS’ instrument^15^).

### Participant recruitment

Clinic records identified 154 households of children who had started routine treatment for MDR-TB when they were below 15 years of age at Tygerberg Hospital between 1 January 2018 and 31 December 2021 and lived in the Cape Town metropolitan area. The study team attempted to contact caregivers by telephone to determine their interest in participating. Upon providing written consent with additional assent from children as applicable, 45 participants (29%) completed the survey in person between March and September 2021. Study staff facilitated participation by collecting participants from their homes or a convenient location. Reasons for not taking part included 11 caregivers (7%) who did not wish to participate, 20 caregivers (13%) who were living out of the area, 9 caregivers (6%) expressed interest but were unable to attend, and 69 caregivers (45%) were uncontactable on the phone numbers available. The reasons and implications of the observed response rate are addressed further in discussion.

### Provincial health data

Information on patients’ health care resource utilisation (such as the number of appointments and hospitalisations) was extracted from public health care records by matching patients’ electronic folder number which serves as a unique identifier. Participants provided their assent and/or consent for researchers to access their electronic health records using this unique identifier through the Provincial Health Data Centre (PHDC). The PHDC, managed by the Western Cape Provincial Government, is an innovative platform integrating diverse per-patient health-system data, including hospital admissions, laboratory results, diagnosis, and primary health care details.^16^ A parallel costing analysis in paediatric MDR-TB also used PHDC to synthesise health-system costs.^17^

### Socio-economic status and income

Household socio-economic data were characterised by matching reported household assets in the South African Demographic and Health Survey (DHS) 2016, enabling derivation of wealth index scores for each household from the DHS wealth index coefficients. The DHS wealth index has been applied in more than 90 low- and middle-income countries globally and is an established composite indicator that incorporates survey responses on factors such as ownership of assets, dwelling characteristics, water access, and sanitation facilities to provide national estimates of relative SES.^18^ For this study, household responses were matched to the DHS wealth index coefficients from the general South African population as well as a sub-sample of urban households in the Western Cape, South Africa, offering both national and localised comparisons (Supplementary Data Appendix). Information on monthly household income was collected directly via the survey. For those respondents who preferred not to provide household income (n = 9), household income was estimated as the median household income of other households in the survey that were in the same SES quintile. Summary statistics of the effect on household income following the COVID-19 pandemic were also recorded.

### Patient costs

Household survey data were also used to estimate the expected costs of accessing routine care, including visits to hospitals and primary health care clinics. Direct household costs of accessing care information reported by participants were combined with individual patient treatment profiles, sourced from the PHDC data. Costs reflected the entire episode of TB care, including diagnosis, acute treatment period, and continuation, and are presented in South African Rand (ZAR) 2021 values (the year of collection) and United States Dollars (USDs), applying the average exchange rate in 2021 (USD 1: ZAR 14.78).^19^ Cost data were summarised using medians and interquartile ranges (IQRs) due to right-skewed distributions, and subgroup comparisons (by age group and disease site) were conducted using the Wilcoxon rank-sum test, with statistical significance defined at *P* < 0.05.

Total household costs related to the child’s episode of TB care were calculated to assess the financial burden of paediatric MDR-TB treatment on households. Catastrophic health expenditure was estimated at the household level using the WHO-recommended threshold of 20% of annual household income, as specified in WHO patient cost survey guidance.^5^ For comparison to other country contexts, household costs were adjusted for purchasing power parity and presented in Supplementary Data Appendix.

### Health-related quality of life

Responses from the HRQoL survey module were analysed to generate summary statistics and estimate the health decrements associated with the different stages of the disease. The TANDI^14^ survey was completed by the survey participant on behalf of the child if the child was between 0 and <4 years of age (n = 17). The EQ-5D-Y^13^ was completed if the child was between 4 and <15 years of age (n = 29).

### Ethical statement

The study received ethics approval from the Stellenbosch University Health Research Ethics Committee (no. N20/09/102).

## RESULTS

Forty-five households and 48 children were included in the survey (Table 1). Three households contributed data for two children each. Most households were female-headed (34 families, 76%) and *isiXhosa*-speaking (28 families, 62%), with 15 (33%) reporting Afrikaans and 2 (4%) other languages spoken at home. Household heads were typically a parent of the child 34 (76%), and 30 (67%) had attended but not completed high school. Formal employment was limited, with only 14 (12%) adults employed in the formal sector and 23 (51%) households reporting no employed adult member. Additional health burdens were common: 14 (31%) households included another person with TB, and 8 (18%) reported someone living with HIV in the household; two children (4%) were living with HIV. Among the 48 children, 25 (48%) were <2 years old at the time of TB treatment initiation, and an additional 15 (33%) were 2 to <5 years. The sample was evenly split by sex. Thirty-seven (77%) children had pulmonary TB, while 17 (35%) had extra-pulmonary disease. Ten of 45 households (22%) reported that a household member discontinued employment due to the child’s MDR-TB illness.

**Table 1.** Characteristics of survey responses of households with children who had multidrug-resistant TB (n = 45 households).

Characteristic–household		Number (% of households)
Household language	isiXhosa	28 (62)
	Afrikaans	15 (33)
	Other**^A^**	2 (4)
Sex of household head	Male	11 (24)
	Female	34 (76)
Age of household head	25 to <30 years	4 (9)
	30 to <40 years	17 (38)
	40 to <50 years	13 (29)
	50 to <60 years	8 (18)
	60+ years	3 (7)
Relationship of patient to household head	Daughter/son	34 (76)
	Grandchild	6 (13)
	Other**^B^**	5 (11)
Highest level of education of household head	Attended but did not complete high school	30 (67)
	Completed high school	12 (27)
	Degree or diploma after completing high school	3 (7)
Employment	Households with no adult member employed	23 (51)
	Proportion of adults in formal employment**^C^**	14/113 (12)
	Total unemployment rate of all adults in the household**^C^**	77/113 (68)
TB/HIV	Someone in the household with TB (in addition to the child)	14 (31)
	Someone in the household living with HIV	8 (18)
Characteristic – child (n = 48)		
Age of child	<2 years	23 (48)
	2 to <5 years	16 (33)
	5 to <15 years	9 (19)
Sex of child	Male	24 (50)
	Female	24 (50)
HIV	HIV positive	2 (4)
Site of TB	Pulmonary	33 (69)
	Extra-pulmonary	15 (31)

A Other = English (n = 1); French (n = 1).

B Other: Niece/nephew: n = 1; neighbour/someone I care for: n = 4.

C Proportion of adults (n = 113) in survey households below 65 years of age and who are not in education.

### Household characteristics

**Figure 1.**
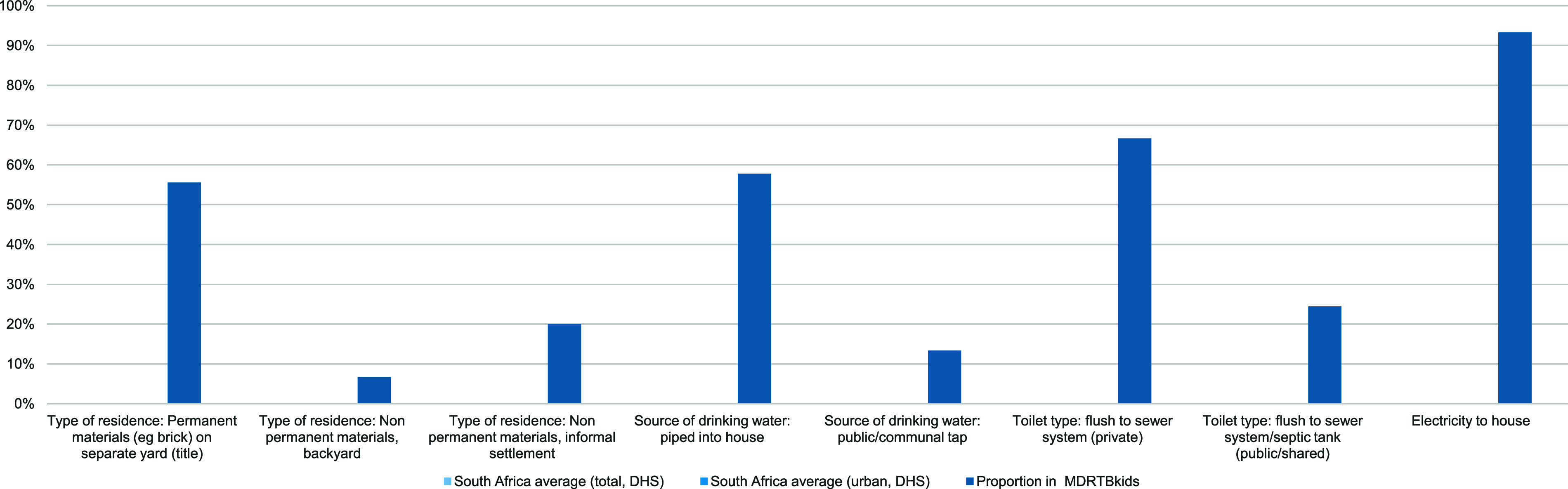
Comparison of household infrastructure in MDRTBkids survey households (n = 45), all South African urban households, and all South African households.

When household infrastructure indicators for the study sample were compared with national and urban South African averages, a lower proportion of MDR-TB households reported access to electricity compared to the national and urban averages (Figure 1). Inadequate housing infrastructure was more common in study households, with 20% residing in informal dwellings compared to 10% of all urban households. Access to piped water inside the dwelling was also lower among study households than the urban average. These findings suggest that households affected by childhood MDR-TB face disproportionate infrastructure disadvantages compared to the broader urban population. COVID-19 had a substantial impact on households, with 24 (53%) reporting that the pandemic had resulted in a major loss of income for the household, and 16 (37%) of caregivers reporting that someone in the house had lost employment due to COVID-19 (Supplementary Data Appendix). Most households (89%) reported receiving at least one form of government social assistance, most commonly the Child Support Grant. In 2021, this means-tested transfer provided ZAR 460 per month (approximately US$31) to the primary caregiver of an eligible child under 18 years of age.

### Costs of accessing care

For children aged < 5 years (n = 39), the median total household cost was ZAR 8,004 (USD 542) (IQR ZAR 4,310–13,728), including direct medical and non-medical costs of median ZAR 1,949 (USD 132) (IQR: 840–3,430), supplementary costs (which included food purchased while attending health facilities and additional food purchased by the household due to the child’s condition) of median ZAR 2,014 (USD 136) (IQR: 300–4,711), and indirect costs related to time and productivity losses of median ZAR 2,959 (USD 200) (IQR: 1,041–4,210) (Table 2). Among children 5 to <15 years (n = 9), the median total household cost was slightly higher at ZAR 9,130 (USD 618) (IQR: 7,370–15,416), with indirect costs accounting for the largest share with median ZAR 4,394 (USD 297) (IQR: 2,917–5,357). The median cost burden as a proportion of household income was 19.4% (IQR: 8%–36%) for households with younger children and 24.9% (IQR: 16%–49%) for those with older children. Overall, the combined sample had a median total household cost of ZAR 8,350 (USD 565) (IQR: 4,507–14,133), equivalent to 20.3% (9%–39%) of reported annual household income.

**Table 2.** Median household cost (ZAR) of MDR-TB treatment for a child (interquartile range) by age range, cost type, and proportion of annual household income (n = 48 children).

Characteristic	Direct costs	Supplementary direct costs	Indirect costs	Total household costs	Total household costs as proportion of annual household income**^A^**	Proportion facing catastrophic health expenditure**^B^**
Age						
0 to <5 years (n = 39)	1,949 (840–3,430)	2,014 (300–4,711)	2,959 (1,041–4,210)	8,004 (4,310–13,728)	19.4% (8%–36%)	47.2%
0 to <2 years (n = 23)	2,986 (1,088–3,479)	1,220 (300–4,976)	3,586 (2,116–5,139)	9,880 (4,870–14,766)	19% (9.2%–32.0%)	47.6%
2 to <5 years (n = 16)	1,050 (588–2,387)	3,093 (322–4,513)	1,845 (976–3,469)	5,540 (2,410–11,219)	19.4% (3.8%–39.0%)	46.7%
5 to <15 years (n = 9)	2,900 (770–4,940)	1,836 (1,248–6,322)	4,394 (2,917–5,357)	9,130 (7,370–15,416)	24.9% (15.5%–48.7%)	66.7%
Site of disease						
Pulmonary TB (n = 33)	1,720 (768–3,060)	1,248 (43–3,293)	2,267 (976–3,920)	6,260 (2,554–9,678)	13.0% (5%–33%)	41.9%
Extra-pulmonary TB (n = 15)	3,300 (1,099–5,881)	5,885 (854–12,109)	4,210 (3,429–6,343)	15,468 (9,880–26,298)	28.9% (19%–51%)	71.4%
Total	2,016 (820–3,589)	1,982 (405–4,844)	3,168 (1,131–4,818)	8,350 (4,507–14,133)	20.3% (8.7%–39.0%)	51.1%

ZAR = South African Rand; MDR-TB = multidrug-resistant TB.

A Mean proportion of annual household income in n = 45 households.

B Proportion households (n = 45) where total household expenditure costs is greater than 20% of annual household income.

Costs were right-skewed, with most households incurring less than ZAR 15,000 (USD 1,015) for the cost of treatment, but a small number reporting much higher expenses. The distribution was approximated by a gamma distribution (shape [α] = 1.52, scale [β] = 6,436) (Supplementary Data Appendix). Costs differed significantly by site of disease (pulmonary or extra-pulmonary); no significant differences in cost were observed by age group.

**Figure 2.**
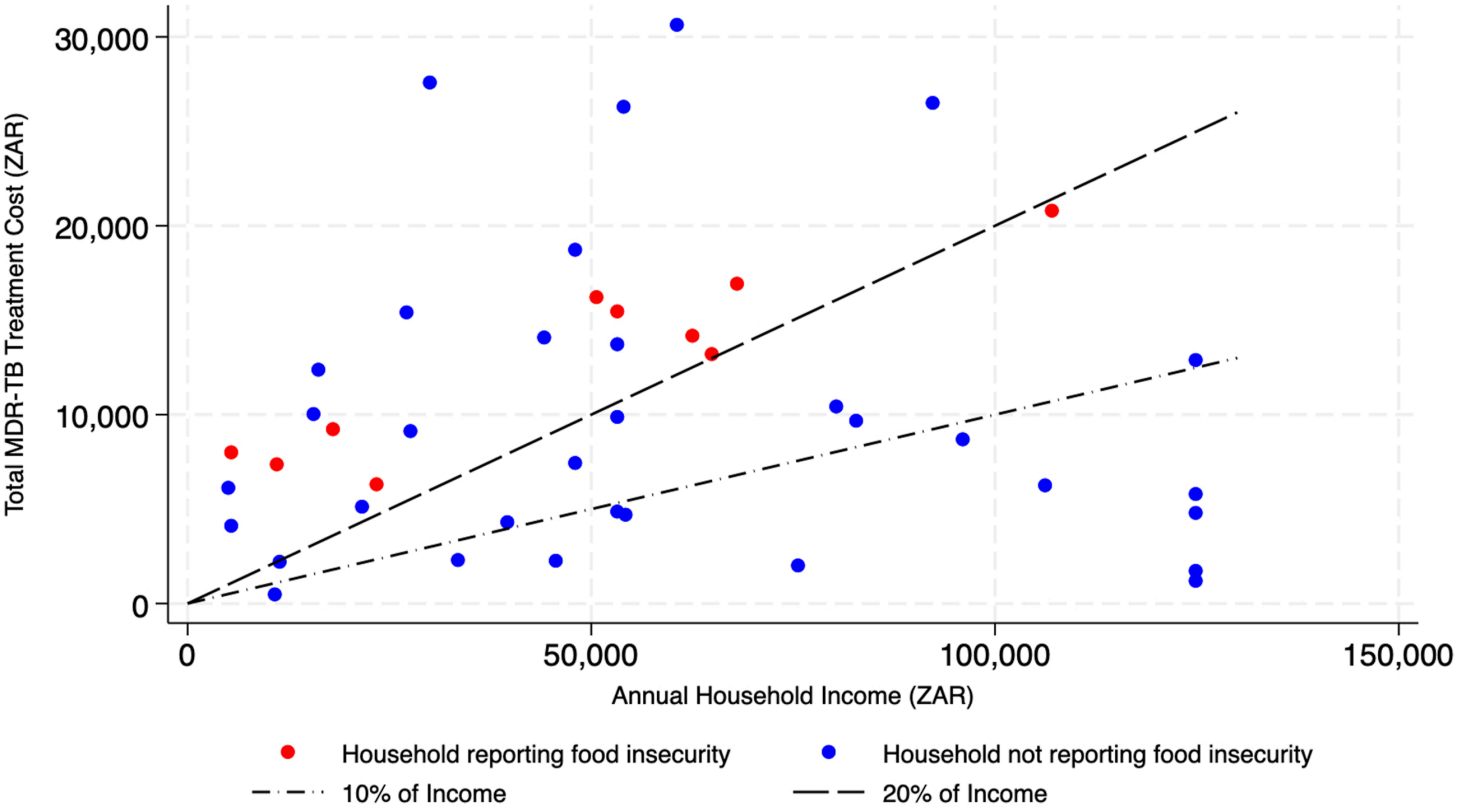
Household costs of accessing multidrug-resistant TB treatment in children compared with annual household income, with 10% and 20% of annual household income affordability thresholds (n = 45 households).

More than half (51.1%) of households incurred treatment costs exceeding 20% of their annual income, a reference threshold for catastrophic health expenditure as defined by the WHO^5^ (Figure 2). Food insecurity, informed by the survey question as ‘has anyone in the household gone to bed hungry in the previous 3 months because there was not enough food’, was strongly associated with catastrophic health expenditure.

### Health-related quality of life

HRQoL was assessed using the EQ-5D-Y in 29 (63%) children and the TANDI instrument in 17 (37%) children. Most children reported no problems across EQ-5D-Y dimensions, with 27 (93%) having no difficulty doing usual activities and 25 (86%) reporting no problems with mobility or feeling worried or sad (Table 3). However, seven (24%) reported some or a lot of pain or discomfort. The median EQ-visual analogue scale (VAS) score was 90 (IQR: 80–100), with a mean of 89 (standard deviation [SD]: 13.1). Among the TANDI sub-sample, problems were most frequently reported in the pain (four children, 24%) and relationships (two children, 12%) dimensions. The median VAS score in this group was slightly higher at 95 (IQR: 80–100), with a mean of 87 (SD: 14.4). These results suggest that while most children experienced few limitations in mobility or self-care, pain and emotional well-being were notable concerns for some. There was not a statistically significant difference in HRQoL scores between extra-pulmonary and pulmonary disease; however, it is expected that this is due to low patient numbers rather than establishing no differences between the groups.

**Table 3.** Health-related quality of life of children in the MDRTBkids survey (n = 46).

	Dimension	Response		
		No problems	Some problems	A lot of problems
EQ-5D-Y (n = 29)	Mobility	86%	10%	3%
	Looking after self	86%	3%	10%
	Doing usual activities	93%	3%	3%
	Having pain or discomfort	76%	14%	10%
	Feeling worried or sad	86%	10%	3%
TANDI (n = 17)	Movement	94%	6%	0%
	Play	94%	0%	6%
	Pain	76%	24%	0%
	Relationships	88%	12%	0%
	Communication	88%	0%	6%
	Eating	94%	0%	6%
		Visual analogue score		
		Median (IQR)	Mean (SD)	Range
EQ-5D-Y (n = 29)		90 (80–100)	89 (13.1)	50–100
TANDI (n = 17)		95 (80–100)	87 (14.4)	50–100

EQ-5D-Y = EuroQol 5-dimension youth version health-related quality of life survey instrument; TANDI = Toddler and Infant health-related quality of life survey instrument; IQR = interquartile range; SD = standard deviation. n = 46 as two respondents did not report HRQoL data.

## DISCUSSION

This study provides one of the first empirical cross-sectional assessments of household SES and the financial burden associated with childhood MDR-TB. It captures the real-world experiences of affected households and their interactions with the health system across multiple dimensions. Importantly, it also offers one of the few available descriptions of HRQoL among children with MDR-TB, particularly in the youngest age groups.

A notable strength of this analysis is its integration of primary survey data with a unique routine electronic health information platform in the Western Cape. This approach allows the household ‘cost per event’ (i.e., cost per hospital or primary health care clinic visit) to be directly reported by caregivers, while the ‘number of events’ is derived from electronic health records. By separating cost and utilisation data sources, the method reduces recall bias and overcomes common limitations of household costing surveys, where respondents often cannot accurately estimate the frequency of health-system interactions across an extended course of treatment. This innovation is particularly relevant for cross-sectional surveys, which otherwise face uncertainty in estimating expected health care utilisation over the full treatment period.

Current WHO guidance on TB patient cost surveys relies primarily on manual data collection from household respondents, with limited reference to the potential use of electronic health records.^5^ While electronic health information systems remain underdeveloped in many high-burden settings, our findings demonstrate that linking routine health records with household-level survey data is both feasible and informative. This provides a new methodological avenue for patient cost studies, with potential to improve precision and reduce data collection burden in contexts where electronic health records are available.

In the study setting, households affected by childhood MDR-TB were disproportionately socio-economically disadvantaged. Compared to national and urban averages from the 2016 DHS, these households had lower rates of formal employment, lower educational attainment, and poorer infrastructure access.^20^ This aligns with global evidence that TB risk is closely linked to structural poverty, but few studies have documented this association specifically in children, particularly in the era of decentralisation for MDR-TB care in provinces such as the Western Cape. Nearly one quarter of households reported that a caregiver stopped working because of the child’s MDR-TB, highlighting the potential for prolonged treatment to disrupt household income and amplify financial vulnerability beyond direct medical and transport costs.

Household costs were not statistically different between age groups. The statistically significant higher household costs observed among children with extra-pulmonary disease compared to pulmonary disease are likely related to greater clinical severity and higher rates of hospitalisation, although the sample size was limited and the analysis was exploratory.

Our findings align with and extend previous research from South Africa. Loveday et al.^21^ reported transport costs of ZAR 93 to ZAR 412 per hospital visit among caregivers of children with MDR-TB, in households earning just ZAR 1,900–2,800 per month. Ramma et al.^22^ found substantial household costs among adults with rifampicin-resistant TB despite care being nominally free. Foster et al.^23^ documented mean total costs of ZAR 4,302 per TB episode and noted that delays in diagnosis were more common among the poorest patients. These studies confirm that TB-related costs disproportionately affect poorer households and support the case for stratifying cost data by SES – an approach adopted in the present study. These data also indicate the importance of social and economic support for families affected by MDR-TB.

This study also represents one of the earliest applications of the EQ-TIPS, formerly known as TANDI (Toddler and Infant) instrument^14^ to assess HRQoL among young children (0–<4 years) with TB. The EQ-5D-Y1^13^ was used in older children (≥4 years), although both tools have limitations in capturing health utility in younger age groups.^24^ In young children, HRQoL assessment relies on proxy reporting by caregivers, which may not fully reflect the child’s internal experience of pain, emotional distress, or well-being. Limited expressive language and cognitive development constrain direct self-report, and it can be challenging to distinguish illness-related limitations in mobility, play, or social interaction from normal variation in developmental stage. The findings point to the need for further development and validation of child-specific HRQoL instruments, including longitudinal assessment in high-TB burden settings.

Our study had several notable limitations. The participation rate as a proportion of caregivers of children who were identified as potentially eligible based on clinic records was 29%, largely attributable to the 45% of participants who could not be contacted by phone. Although household mobile phone coverage in South Africa is high,^25^ maintaining contact and linkage to care with patients and families undergoing TB remains challenging, with out-of-date or disconnected phone numbers being a major cause.^26^ The study was not resourced or approved to undertake unscheduled home visits either directly or accompanying ward-based outreach teams or community health workers. This incomplete tracing introduces the possibility of selection bias. It is plausible that households who could not be contacted were more socio-economically vulnerable, for instance, if financial instability is associated with more frequent changes in contact details. In that case, the financial burden observed in this study underestimates the true burden among all families affected by childhood MDR-TB. Conversely, it is also possible that changes in phone numbers occur across socio-economic strata in the potentially eligible sample. In addition, 13% of potentially eligible households were not included as they were living out of the local area. This limited inclusion of households residing in more distant urban or semi-rural areas. The rate of those who expressed interest but could not attend (6%) or who did not want to participate (7%) was in line with expectations and is not expected to have introduced significant bias. While the direction and magnitude of sampling bias cannot be determined with certainty, the findings should therefore be interpreted as reflecting the experiences of contactable households within this urban referral population rather than all eligible families. The survey was conducted during and shortly after the COVID-19 pandemic, a period associated with substantial economic disruption in South Africa, particularly among households dependent on informal employment. Over half of participating households reported major income loss during this period. It is therefore possible that the relative burden of MDR-TB-related costs, when expressed as a proportion of household income, was amplified by pandemic-related income shocks. However, the principal drivers of household expenditure identified in this study, including transport, time costs, and hospital-related visits, are structural features of MDR-TB care that are unlikely to change substantially in a post-pandemic setting. The findings should therefore be interpreted within the context of pandemic-affected income levels, while recognising that the underlying mechanisms of financial vulnerability remain relevant beyond the immediate COVID-19 period. Because the study population reflects households accessing public-sector MDR-TB services, the findings are most directly applicable to similar public-sector settings and should not be assumed to represent the experiences of higher-income households or private-sector care pathways. However, given that MDR-TB care in children is predominantly delivered through the public sector in South Africa, the findings remain highly relevant to the majority of affected households. In terms of methodological limitations, treatment cost estimates were based on observed prices and self-reported utilisation, which may not capture all informal or unrecorded expenditures. In addition, time costs were valued using the national minimum wage, which may not fully reflect opportunity costs across different employment contexts. Lastly, the HRQoL data were collected in a relatively small cohort without a healthy control group, which limits the ability to determine empirical QoL decrements from this analysis; however, it does provide an important foundation for further research in this population.

The study reinforces the substantial economic and social burden of childhood MDR-TB on already vulnerable households. Even when treatment is provided free of charge, indirect and non-medical costs can lead to catastrophic health expenditure. The study was not designed to establish causal mechanisms to determine whether household poverty increases likelihood of childhood MDR-TB, or whether having a child in the household with MDR-TB increases the likelihood of poverty. The nature of childhood MDR-TB as an expected cause, and symptom, of poverty reflects the complex dynamic between social environment and health. The analysis should be interpreted as descriptive and hypothesis-generating, and that larger studies would be required to robustly examine determinants of catastrophic health expenditure. These findings reinforce the need to embed TB services within a broader social protection and health-systems framework. This may include a range of services including nutrition, transport and financial support, and psychosocial support. In practical terms, this could include interventions such as targeted transport vouchers for families required to attend frequent clinic or hospital visits, nutritional supplementation for children undergoing prolonged treatment, and streamlined linkage to existing social grants for eligible households. Given that indirect costs, particularly caregiver time and income disruption, accounted for a substantial proportion of total household burden, interventions that reduce the frequency or duration of facility-based visits may also mitigate financial impact. Strengthening coordination between TB programmes and social development services may therefore represent a pragmatic approach to reducing catastrophic health expenditure among affected households. In line with the WHO’s End TB Strategy, targeted mechanisms are needed to identify and support vulnerable households, especially given the disproportionate burden of childhood TB among socio-economically disadvantaged populations.

## CONCLUSION

Childhood MDR-TB imposes a substantial financial burden on already socio-economically vulnerable households in this urban South African setting, with over half experiencing catastrophic total costs despite treatment being provided free of charge. Indirect costs, particularly caregiver time and employment disruption, were major contributors to household economic strain, and costs were significantly higher among children with extra-pulmonary disease. Although HRQoL was generally reported as high, measurement in very young children remains methodologically challenging and warrants further validation. Integrating TB services with social protection mechanisms may be essential to reducing catastrophic costs and advancing the WHO End TB Strategy target of eliminating financial hardship due to TB.

## Supplementary Material
